# Application of exosomal miRNA mediated macrophage polarization in colorectal cancer: Current progress and challenges

**DOI:** 10.32604/or.2023.043481

**Published:** 2023-11-15

**Authors:** YUN ZHANG, SHALING TANG, YUBO GAO, ZHONGTING LU, YUAN YANG, JING CHEN, TAO LI

**Affiliations:** 1 Department of Oncology, School of Clinical Medicine, Ningxia Medical University, Yinchuan, China; 2 General Hospital of Ningxia Medical University, Yinchuan, China; 3 School of Basic Medical Sciences, Ningxia Medical University, Yinchuan, China; 4 Department of Surgical Oncology, Tumor Hospital, The General Hospital of Ningxia Medical University, Yinchuan, China

**Keywords:** Colorectal cancer, Exosomes, microRNA, Macrophages, Treatment

## Abstract

Colorectal cancer (CRC) is a major global health problem with high morbidity and mortality rates. Surgical resection is the main treatment for early-stage CRC, but detecting it early is challenging. Therefore, effective therapeutic targets for advanced patients are still lacking. Exosomes, tiny vesicles in body fluids, play a crucial role in tumor metastasis, immune regulation, and drug resistance. Interestingly, they can even serve as a biomarker for cancer diagnosis and prognosis. Studies have shown that exosomes can carry miRNA, mediate the polarization of M1/M2 macrophages, promote the proliferation and metastasis of cancer cells, and affect the prognosis of CRC. Since the gastrointestinal tract has many macrophages, understanding the mechanism behind exosomal miRNA-mediated macrophage polarization in CRC treatment is crucial. This article summarizes recent advancements in the study of exosomal miRNAs in CRC and their potential as diagnostic and prognostic markers.

## Introduction

Malignant tumors have always been the greatest threat to human survival. Colorectal cancer is the third most common cancer worldwide and one of the most common digestive system tumors. According to statistics, colorectal cancer (CRC) accounts for 10% of all malignant tumor incidence, and more than 900,000 people have died from CRC [1]. The pathogenesis of CRC is complicated.

The current treatment of choice for most early-stage colorectal cancers is surgical resection, and systemic treatment (chemotherapy, targeted therapy, and immunotherapy) is used for unresectable metastatic colorectal cancer. Since this disease is characterized by slow progression, most patients are found to be in stage II or III and may miss the optimal treatment period [2]. It has been demonstrated that some metastatic colorectal cancer patients have improved survival, and overall survival (OS) has exceeded 2 years in some patients after combination therapy [3]. In particular, personalized treatment plans based on the molecular and pathological characteristics of the tumor can effectively improve patient survival [4]. Despite some progress in tumor precision therapy, the 5-year survival rate does not exceed 20% in patients with stage IV colorectal cancer [2,5].

Exosomes are involved in various physiological and pathological processes in the human body and are widely recognized as an essential connection point for intercellular communication [6,7]. An increasing number of studies have shown that exosomes derived from cancer can directly or indirectly affect the immune cells and participate in the occurrence and development of tumors [8]. It is well known that immune cells play an enormous part in anti-tumor therapy. Generating specific T cells that can effectively target tumor cells and ensure the induction of a long-term anti-tumor protective immune response is the current focus of immunotherapy against cancer [9]. Macrophages are one of the major immune cells in the human body. They can be activated by different forms of stimulation, differentiate into different phenotypes, and perform different functions, a complete process known as “macrophage polarization” [10]. Massive infiltration of tumor-associated macrophages(TAMs) in CRC tissues, which not only promotes tumor growth by inhibiting anti-tumor immunity, stimulating angiogenesis and tissue remodeling but also is closely related to metastasis and drug resistance of colorectal cancer, which is one of the important reasons for poor prognosis in CRC patients [11–13]. Most notably, exosomes secreted by tumors can carry miRNAs and transfer them to macrophages, thus regulating the polarization of macrophages through certain pathways to further affect the progression of tumor cells [14]. As a matter of fact, this article discusses the application of exosomal miRNA-mediated macrophage polarization in CRC, hoping to provide an effective strategy for the future immunotherapy of CRC and the development of anti-tumor drugs.

## Biological Functions and Roles of Exosomal miRNAs in Colorectal Cancer

### Biological functions of exosomal miRNAs

**Figure 1:**
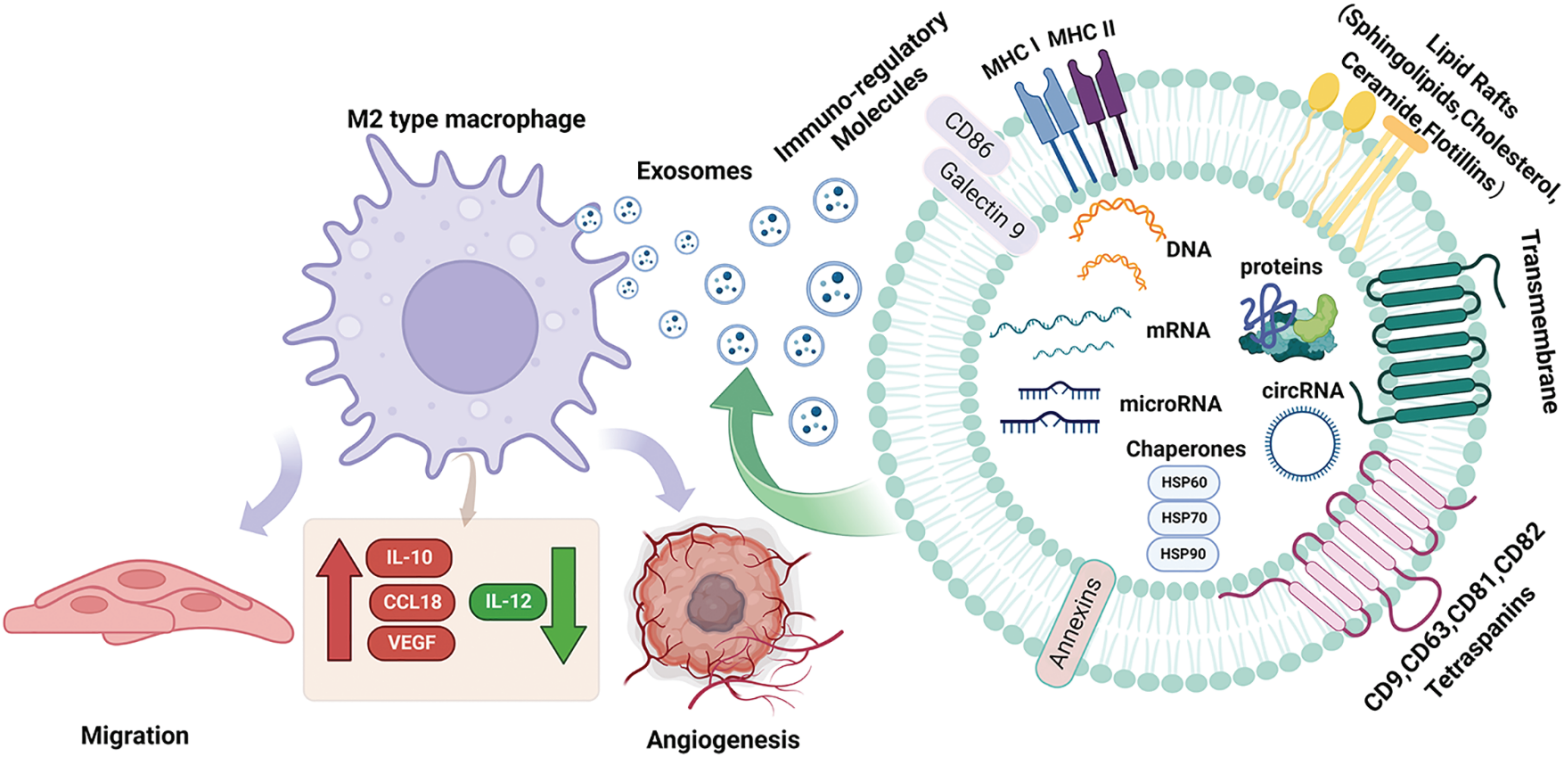
Structural characteristics of exosomes and their components. Exosomes mediate the polarization of M2-type macrophages on tumor cells. Exosomes are produced by vesicles, nucleic acids (miRNA, DNA, microRNA), lipids and proteins (cytoplasmic proteins, tetradonins and membrane receptors) and other goods are absorbed by cells. When they fuse with the cell membrane, exosomes are released into the extracellular space and arrive at the designated site, which causes macrophages to undergo M2 polarization, resulting in IL-10. CCL18, VEGF increased, IL-12 decreased, decreased immune response, increased tumor metastasis and angiogenesis; Acting on M2 macrophages increases IL-10, CCL18, VEGF, decreases IL-12, reduces immune response, and increases tumor metastasis and angiogenesis. CCL18, Chemokine Ccmotif Ligand 18; circRNA, circular Ribonucleic Acid; DNA, deoxyribonucleic acid; HSP60, heat shock protein 60; HSP70, heat shock protein 70; HSP90, heat shock protein 90; IL-10, interleukin-10; IL-12, interleukin-12; microRNA, micro ribonucleic acid; MHC I, major histocompatibility complex I; MHC II, major histocompatibility complex II; VEGF, vascular endothelial growth factor.

Exosomes are tiny vesicles wrapped in bimolecular lipid membranes and secreted by cells, originally discovered by Harding [15]. It should be noted that exosomes do not act directly on the tumor, but have an effect on the tumor by releasing internal substances. Its interior contains a variety of substances inside the exosome such as proteins, circRNAs, miRNAs, and lncRNAs. MiRNAs are expressed at the highest level among multiple RNAs in exosome [16]. MicroRNAs (miRNAs) are small endogenous RNAs that are transcribed by tumor cells in the nucleus to form precursor miRNAs. The precursor miRNAs are transported from the nucleus to the cytoplasm, and then enter the body formed by a large vesicle enveloping a small vesicle during lysosome generation. The multivesicular body fuses with the cytoplasmic membrane and is released into the extracellular space. Exosome-derived miRNAs are formed and then transported to the corresponding receptors to participate in the pathological and physiological processes of the body [17,18]. It is not only a carrier of intercellular signal transduction, involved in signaling cell proliferation and differentiation, apoptosis, and transduction pathways but also plays a vital role in processes such as immune response [19]. Compared with other exosomal RNAs, exosome-derived miRNAs have the following advantages: firstly, they have high stability and can be easily detected in various tissues and body fluids, and some miRNAs form complexes with proteins that can remain active and stable in circulation to prevent degradation; secondly, they act as an efficient carrier to participate in various regulatory activities of the body, regulating the activities of target cells through transport, and it exerts anti-apoptotic, pro-proliferative, and angiogenic effects, inducing cell differentiation, and regulating immunity [20,21]; More importantly, miRNA regulation can also alter macrophage phenotype and cytokine expression, activating related signaling pathways leading to tumorigenesis [22] (Fig. 1). Therefore, the role of exosomal miRNAs in tumor cells cannot be ignored.

### Role of exosomal miRNAs in the invasion and metastasis of colorectal cancer

Proliferation a key manifestation of cancer cell proliferation, which can be observed through changes in the expression or activity of cell cycle-related proteins [23]. Multiple studies have confirmed the involvement of exosomal miRNAs in cancer development and progression through various pathways and signaling activation [24]. Overexpression miR-155-5p in M2 macrophage-derived exosomes promotes CRC cell proliferation, antigenic capacity, enhanced CD3 T cell and IFN-γ T cell proliferation, and significantly reduces IL-6 expression levels, leading to colon cancer the immune escape and induction of tumor formation [25]. The research by Zhang et al. [26] reported that miR-183-5p levels in [27] M2-TAM-derived exosomes in colon cancer (CC) were significantly increased, and the down-regulation reversed the tumor-promoting effect mediated by M2-TAM. The further mechanism study found that the exosome miR-183-5p accelerated the progress of CC by mediating the AKT/NF-κB pathway and activating it. Similarly, CRC cell-derived exosomes carrying miR-1249-5p, miR-6737-5p, and miR-6819-5p have been shown to inhibit TP53 expression in fibroblasts and accelerate CRC progression [27]. In addition, Li et al. found that has-miR-6825-5p was competitively bound to upregulate CXCR3 expression, leading to M2 polarization and promoting the progression of neuroendocrine differentiated CRC [28]. All the above results show that exosomes of cancer cell origin play a key role in the progression of CRC and are highly beneficial for the development of novel therapeutic strategies for CRC.

### The role of exosomal miRNAs in immune regulation of colorectal cancer

It is well known that the immune system cannot be ignored in the process of tumorigenesis and development, and tumor cells must avoid the surveillance of immune cells or inhibit the relevant immune response to avoid being recognized and killed by the immune system when metastasis occurs [29]. miRNA is an effective regulator of immune cell function. Its effect on immune cells extends beyond enhancing the chemotaxis and phagocytosis of neutrophils but also can reduce anti-tumor immune response by regulating the expression of immunomodulatory molecules in tumors and immune cells, thus promoting tumor immune escape [29]. Macrophages, as one of the representatives of innate immune cells, both M1 and M2 macrophages can transform into each other to perform specific biological functions during the immune regulation of the body [30]. Yang et al. argued [31] that high expressions of exosomal miR-106b in plasma were significantly associated with the malignant progression of CRC. Increased levels of exosomal miR-106b contributed to M2 polarization of macrophages through direct inhibition of programmed cell death 4 activation of rapamycin PI3Kγ/AKT. Both studies have revealed a new mechanism of CRC progression and provided potential targets for CRC therapy. Similarly, Zhao et al. reported [32] that the expression level of the exocrine miRNA-320a from CAFs was reduced after being treated with inhibitors. Mechanically, macrophages were polarized into M2 phenotype by regulating PTEN/PI3Kγ signal transduction, which accelerated the malignant behavior of pancreatic cancer cells. On the contrary, Li et al. found [33] that in gastric cancer (GC), the M1 macrophage-derived exosome miR-16-5p triggered T cells to generate an immune response by targeting programmed cell death protein 1 (PD-L1), and the exosome inhibited the progress of GC by reducing the expression of PD-L1. In addition, pancreatic cancer-derived exosomes transfer miRNAs into dendritic cells (DC) and inhibit the expression of RFXAP, thereby reducing the expression of MHC II and inducing immune tolerance of DC [34]. Tumor cells inhibit the maturation and differentiation of immune cells through various signal transduction pathways mediated by exosome miRNA, thus creating an immune microenvironment suitable for tumor growth [35]. Therefore, immune miRNAs may be a valuable tool in the treatment of cancer, helping to assess a patient’s current immune status and develop a rational treatment plan.

### The role of exosomal miRNAs in drug resistance in colorectal cancer

The development of drug resistance in tumor cells is one of the major causes of poorer patient prognosis and the pathways and mechanisms through which drug resistance develops vary among cancers. Tumor stem cells express CD44+ and CD24+ markers on the cell surface and form during Epithelial–mesenchymal transition (EMT) [36]. Cancer stem cells (CSCs) can induce chemotherapy resistance through the DNA damage response pathway. When drug-induced DNA damage is occurs, DNA repair signals act on the cell cycle, leading cell cycle arrest in the G1/G2 phase. Not only that, CSCs can also act on drug-resistance-related signaling pathways [37,38]. In CRC, circulating exosomes contianing miR-100, miR-92a, miR-16, miR-30e, and miR-144-5p were reported as oxaliplatin-based chemoresistance biomarkers [39], and targeting these miRNAs may significantly reduce the development of drug resistance in patients. Similarly, In a mechanistic study, Zhang et al. [40] found that exosomal miRNAs derived from CAFs are also key players in CRC chemoresistance. Exosomal miR-625-3p derived from CAFs may promote the migration and invasion of CRC cells, EMT, and chemoresistance by inhibiting the CELF2/WWOX pathway, which provides an effective strategy for the treatment of CRC. Furthermore, exosomal miR-1247-3p from hepatocellular carcinoma cells induced an activated ecotone of CAF in lung pre-metastatic fibroblasts, leading to the activation of NF-κB signaling, importantly, exosomes led to the resistance of tumor cells to sorafenib treatment [41]. In addition, CAFS-derived exosome miR-106b is also associated with gemcitabine resistance in pancreatic cancer [42]. In conclusion, released exosomal miRNAs can affect drug resistance in tumor patients through direct or indirect pathways, but different miRNAs have different functions, which require further exploration and practice by researchers.

Moreover, low pH value is a specific marker of malignant tumors, which may affect the release and uptake of exosomes by cancer cells. It is suggested that since the acidic microenvironment of tumors is a powerful chemoresistance factor, exosomes may take up molecules in an acidic environment to better fuse with target cells and thus perform a wide range of biological functions [43]. More importantly, exosomes obtained under physiological pH conditions are more likely to be delivered to acidic chambers, avoiding serious side effects of chemotherapeutic drugs [44]. In summary, exosomal miRNA is involved in the progress, immune regulation, and drug resistance of digestive tract tumors, which fully shows the great potential of exosomes in the treatment of digestive tract tumors.

## Effect of Macrophage Polarization on CRC

### Macrophage polarization

**Figure 2:**
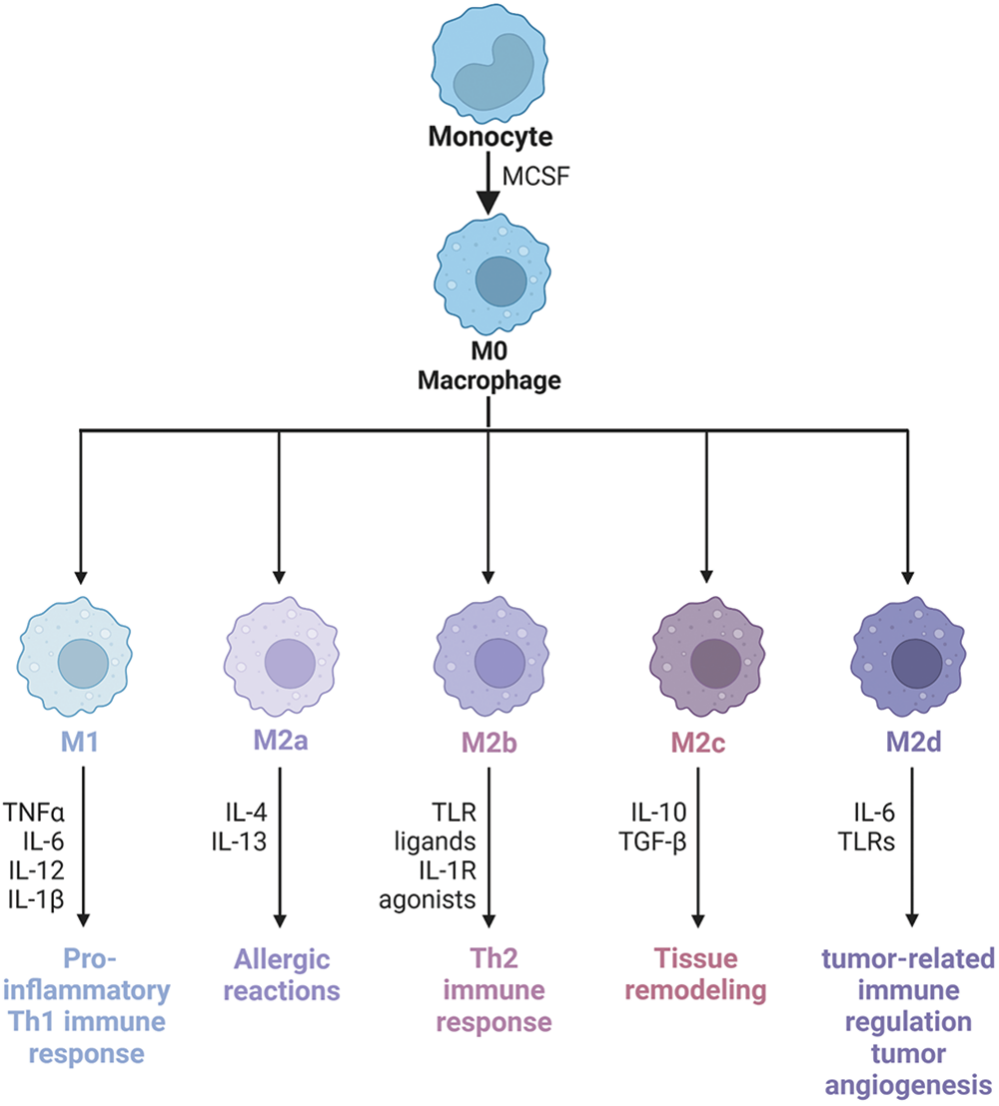
Macrophage phenotype and polarization. Macrophages have good plasticity and can differentiate into different phenotypes under different induced stimuli, mainly M1 and M2. M1 macrophages can cause a strong inflammatory response, on the contrary, M2 macrophages secrete a large number of anti-inflammatory cytokines, which inhibit the inflammatory response. M2-type macrophages activate STAT6 through the IL-4 receptor (IL-4α), thus being polarized by Th2-type cytokines IL-4 and IL-13. Other cytokines can also regulate M2 polarization by activating STAT3 through the IL-10 receptor (IL-10R). M2 macrophage has the characteristics of inhibiting inflammatory reactions, eliminating apoptotic cells, promoting tissue repair and wound healing, improving immune regulation, and promoting angiogenesis. CD206 and CD163 are the most commonly used molecular markers to identify M2-type TAMs in tumor tissues. According to the types of activating factors, M2-type macrophages can be divided into four subtypes: M2a, M2b, M2c, and M2d. M2a macrophages, also known as wound healing macrophages, are induced by IL-4 and IL-13 and play an anti-inflammatory role and tissue remodeling. M2b macrophages are regulatory macrophages, which are induced to differentiate by lipopolysaccharide (LPS) and immune complex, play a role in Th2 activation, secrete high levels of IL-10 and low levels of IL-12, and promote tumor development by weakening immune response and inflammatory response in cancer. M2c macrophages are induced to differentiate by IL-10, transforming growth factor (TGF)-β and glucocorticoid, and release a large amount of IL-10 and promote fibrosis, showing a strong anti-inflammatory effect. M2d macrophages have the characteristics of tumor-like macrophages, which may be involved in tumor-related immunoregulation, tumor growth, and tumor angiogenesis, and are induced by IL-6. IL-1R, Interleukin-1 Receptor; IL-1β, Interleukin-1β; IL-4, Interleukin-4; IL-6, Interleukin-6; IL-10, Interleukin-10; IL-12, Interleukin-12; IL-13, Interleukin-13; Th2, Helper T cell 2; TLR, Toll-like receptor; TLRs, Toll-like receptors; TNFα, tumour necrosis factor; MCSF, macrophage colony-stimulating factor.

Macrophages come from two sources: monocyte-derived macrophages (MDMs) and tissue-resident macrophages (TRMs) [45]. However, the functions of macrophages from two different sources are different. Recently, using single-cell sequencing data sets, the Miriam Merad team obtained samples of untreated NSCLC patients. They found, after modeling mice, that TRMs create a favorable survival environment and stimulate the invasive potential of tumor cells to evade killer T cell attack [46]. However, MDMs contain a wide range of phenotypic states. Macrophages originate from progenitor cells in the bone marrow. They are a type of phagocytes that differentiate from blood monocytes which enter blood vessels and are the body’s first line of defense against pathogenic damage to tissues. Traditionally, they can be divided into classically activated M1 macrophages and alternatively activated M2 macrophages [47]. Generally, the two types of macrophages coexist in the tumor [30]. Macrophage phenotypes are distinguished by surface markers. The interconversion of different phenotypes under specific conditions is known as “macrophage polarization” (Fig. 2). Extrinsic polarization is the main method of macrophage polarization. By secreting different substances to drive macrophages to different phenotypes, for example, cell secretion of IFN-γ will drive macrophages to M1 phenotype polarization, while cell secretion of IL-4 and IL-13 will drive M2 phenotype polarization [48,49]. Second, hypoxia may also be a key driver of macrophage polarization, as well as intrinsic polarization, which is the origin of macrophages, but this is highly controversial because the macrophage population can completely return from the bone marrow origin during lethal chemoradiotherapy [10,50]. M1 macrophages promote anti-tumor immune response by regulating antigen presentation and secreting pro-inflammatory factors, while M2 macrophages exert anti-inflammatory and immunosuppressive effects to promote tumor development [51].

### Effect of M1-type macrophage polarization on CRC

Studies have shown that using exosomes to promote M1-polarization and reprogramming TAMs into M1-like macrophages can significantly increase anti-tumor immunity and inhibit CRC cell growth [52]. The inflammatory environment has long played an important role in the development and progression of colorectal cancer, not only increasing the mutation rate of DNA but also promoting epigenetic changes [53,54]. In the early stage, M1-type macrophages can produce antigen-presenting molecules and activate type I T cells, produce a large number of inflammatory factors, activate immunogenic CD8+ T lymphocytes and natural killer cells, and produce strong anti-tumor effects [55,56]. Bader et al. [57] concluded that clodronate liposomes have a significant effect on reducing colorectal tumor growth, which may be related to the reduction of M1 and M2 macrophage markers and chemokines in colonic tissue. In exploring the mechanism of muciniphila on colorectal carcinogenesis, the investigators found that CRC patients with a significantly reduced abundance of *A. muciniphila* promoted enrichment of M1-like macrophages and produced strong tumor suppressive effects [58]. Similarly, Cheng et al. first demonstrated [59] that protein kinase C α acts as a tumor suppressor in mice by mediating the MKK3/6-P38 signaling pathway thereby promoting IL-12/granulocyte-macrophage colony-stimulating factor-mediated M1 polarization and inhibiting colon tumor growth in mice *in vivo*. Furthermore, the number of TAMs has been shown for many years to correlate significantly with the depth of tumor infiltration, lymph node metastasis, and tumor stage, and may serve as an independent prognostic factor for patients with colorectal cancer [60,61]. Notably, phospholipase D4 in TAMs helps to promote the activation of M1 macrophages, which have a significant antitumor effect on colon cancer cells [62]. Thus, it seems that M1 macrophages act like defense fighters of the body against microbial infections and exert a strong toxic effect on tumor cells in the early or receding stages of the tumor.

### Effect of M2-type macrophage polarization on CRC

Many studies have shown that TAMs usually have an M2 phenotype and are involved in tumor progression [63]. M2 macrophages can suppress the function of CD8+ T cells or promote tumor progression by recruiting regulatory T cells. In advanced tumor stages, most macrophages are more inclined to differentiate into an M2-like state. More importantly, high levels of M2 macrophage infiltration are associated with poor prognosis in CRC patients [64–66].

M2 macrophages produce epidermal growth factor, long fibroblast growth factor-1, vascular endothelial growth factor (VEGF), and high levels of Mitochondrial Membrane Potential (MMP), especially MMP-9, which promotes ECM degradation and tumor angiogenesis, greatly enhancing the invasiveness of colorectal cancer cells [67]. Schäfer et al. concluded [68] that when CT-26 (CRC cells) were co-cultured with RAW264.7 (macrophages), macrophage colony-stimulating factor could be secreted, chemotactic macrophages infiltrated into tumor tissues, enhanced the migration and anti-apoptotic ability of intestinal epithelium, and induced macrophages to differentiate into M2 type. Notably, after EMT-programmed CRC cells activate the expression of cytokines such as IL-4 and CCL2 in cancer cells, these cytokines also enhance the M2-like polarization of macrophages, thus initiating the tumor process [69]. Additionally, cytoplasmic polyadenylation element-binding protein 3 has been shown to exhibit tumor-suppressive effects in CRC. Zhong et al. [70] reported that the knock-down of CPEB3 in CRC cells resulted in an increase in the number of M2 macrophages, and M2 polarization significantly enhanced the proliferation and invasion of CRC cells by targeting IL-6 and activating IL-6R/STAT3 pathway. Oligosaccharide nucleic acids promote CRC progression by regulating the Mir-650/ANXA2 axis and enhance oxaliplatin resistance in CRC cells by polarizing M2 macrophages [71]. Furthermore, the research established that clodronic acid has some effect on reducing colorectal tumor growth, which may be related to the reduction of macrophage markers and chemokines in colon tissue and the reduction of transcription factors associated with CRC [57].

In summary, M2 macrophages play an important role in tumor progression in the later stage of tumor development, and we speculated that M2 macrophages may be effective in reducing tumor growth after removal. In addition, intracellular macrophages are not only associated with poor clinical prognosis but also closely related to chemotherapy resistance. Therefore, macrophage polarization has broad prospects for the treatment and improvement of the prognosis of CRC patients.

## Exosomal miRNAs Mediate Macrophage Polarization to Affect CRC

### Exosomal miRNAs influence CRC by mediating macrophage polarization through the PI3K/AKT signaling pathway

**Figure 3:**
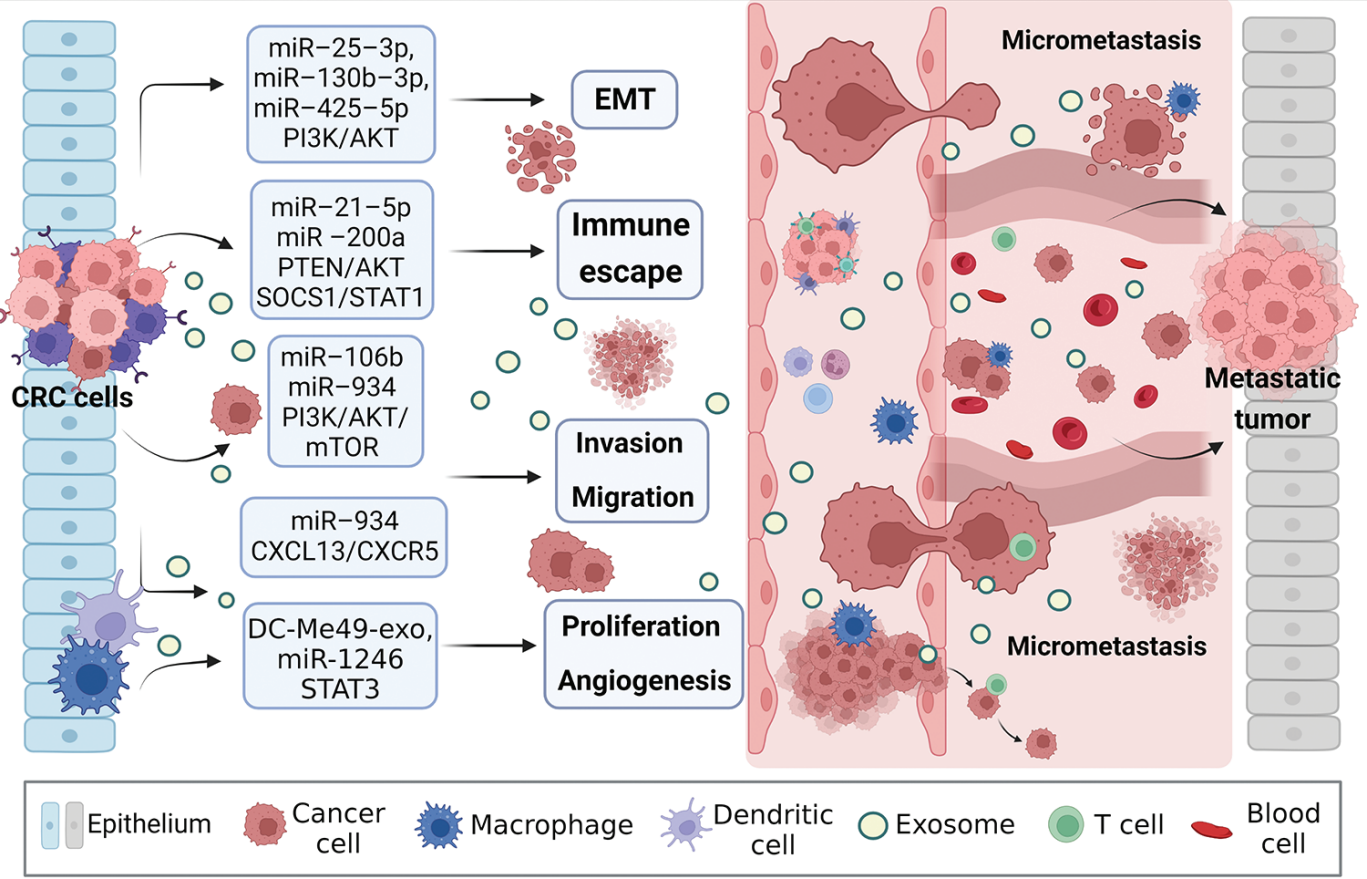
Exosomal miRNAs mediate macrophages to promote CRC metastasis. Activated CRC-derived exosomal miRNAs and other exosomal miRNAs from other sources affect macrophage polarization or other types of cell changes through PTEN/AKT, SOCS1/SATA1, PI3K/AKT/mTOR, STAT3, and other signaling pathways to promote tumor cells to produce EMT, regulate immune escape, cell proliferation, migration, and angiogenesis, create the generation of CRC microenvironment, and stimulate the invasion potential of tumor cells. Avoid being attacked by killer cells, ultimately promoting near and distant metastasis of the tumor. EMT, epithelial-mesenchymal transition.

Exosomal miRNAs can mediate macrophage polarization and impact CRC progression (Fig. 3). The PI3K/AKT pathway is widely present in cells and regulates various biological behaviors. When aberrantly activated, this pathway plays a crucial role in promoting tumor cell proliferation. PI3K mutations are one of the most common types in CRC [72]. A study showed that exosomal miRNAs (miR-25-3p, miR-130b-3p, miR-425-5p) from CRC patients significantly promote EMT and VEGF by activating PI3K/AKT signaling pathway, leading to PTEN-induced macrophage M2 polarization and colorectal cancer liver metastasis [73]. Signal transducer and activator of transcription 1 (STAT1), the first member of the STAT family, is generally considered a tumor suppressor due to its ability to resist pathogen infection and its vital role in the immune response [74,75]. The effects of STAT1 on CRC are gradually being reported. Interestingly, CRC cells release exosomal miR-21-5p and miR-200a, which not only regulate PTEN/AKT but also the SOCS1/STAT1 signaling pathway, significantly inhibiting TAMs-mediated expression of CD8+ T cells [76].

Zhao et al. [77] found that exosomal miR-934 transfer from CRC cells to macrophages downregulates PTEN expression and activate PI3K/AKT signaling pathway, inducing M2 macrophage polarization. This creates a local inflammatory microenvironment, maximizing CRC development and progression. After an in-depth study, it was discovered that polarized M2 macrophages further initiate the CXCL13/CXCR5/NFκB/p65 positive feedback loop in CRC cells by secreting CXCL13. Similarly, another researcher, to assess the ability of EMT CRC cells secreted exosomes and normal CRC cells secreted exosomes to culture macrophages *in vitro*, found that the surface M2 markers (CD163, CD206) and IL-10 increased significantly, while M1 markers (HLA-DR, IL-1b) and IL-12 decreased. On the one hand, EMT-Exos-derived miR-106b further triggered CRC metastasis by suppressing programmed cell death protein-4 (PDCD4) expression in macrophages, thereby activating the PI3K/AKT/mTOR signaling pathway and inducing M2 macrophage polarization [31].

### Exosomal miRNAs influence CRC by mediating macrophage polarization through the JAK/STAT signaling pathway

The JAK-STAT pathway is considered a central player in inflammation-mediated tumorigenesis. The Janus kinase family consists of four main members JAK1, JAK2, JAK3, and Tyk2 [78,79]. The JAK-STAT3 signaling pathway plays an important role in regulating cell proliferation, differentiation, apoptosis, and immunity [80]. The STAT family includes seven members and they play essential roles in many cellular functions [81]. The signal transducer and activator of transcription 3 (STAT3) is a cytoplasmic transcription factor that is central to the regulation of antitumor immune responses and is one of the most studied members. STAT3 not only determines the initial T cell differentiation regulatory and inflammatory T cell lineage, but also controls cell growth, apoptosis, and inflammatory gene transcription, promoting the development of chronic inflammation [82]. Aberrantly activated STAT3 is thought to be a critical initiation site in numerous human cancers [83]. New pathogen-based exosomes have been developed for cancer therapy in recent years, The exosome DC-Me49-Exo, isolated from Toxoplasma gondii-infected dendritic cells, was found to have antitumor properties. Notably, the exosomes suppressed bone marrow-derived suppressor cells (MDSCs) by inhibiting the transcriptional STAT3 signaling pathway. This suggests that exosomes from DCs could be used as a therapeutic strategy for novel CRC by reducing the number of MDSCs [84]. In addition, STAT3 regulates the nuclear localization of β-catenin, which further promotes colitis-associated tumor growth [85].

Another member of the STAT family, STAT6, has also been gradually reported on its impact on CRC. Studies have demonstrated that STAT6 plays a mojar role in IL-4 and IL-13-induced EMT and CRC cell invasiveness. Targeting STAT6 signaling can inhibit tumor growth and metastasis as well [86]. Recently, Sushrut’s team identified for the first time a specific exosome that targets antisense oligonucleotides controlling the macrophage phenotype, leading to greatly enhanced potency of STAT6 expression, effectively inducing reprogramming of TAMs to the M1 phenotype and generating an efficient immune response that sustains TME in a pro-inflammatory and anti-tumor state [87].

All of the above studies suggest that exosomal miRNAs can influence the proliferation, invasion, and metastasis of CRC by regulating the balance between M1 or M2 macrophages, which is highly correlated with the prognosis of tumor patients.

## Prospects and Challenges of Exosomal miRNAs in CRC Diagnosis, Prognosis and Treatment

Exosome-derived miRNAs play a crucial role in CRC and have promising clinical applications. According to current studies, there are four main applications: diagnostic and prognostic markers, therapeutic targets, promotion of metastatic invasion, and chemotherapy resistance.

In general, the sensitivity and specificity of blood-based screening tumor markers are low, and there is no marker with 100% tumor specificity. However, exosomal miRNAs have shown great potential for the early detection of CRC diagnosis. Firstly, its sensitivity and specificity are significantly higher than those of carcinoembryonic antigen and carbohydrate antigen 19-9 [88], and secondly, exosomes are present in body fluids and are more convenient and non-invasive to take relevant tests than endoscopic biopsies for pathological examination [89]. In 2015, Matsumura et al. [90] identified exosomal miR-19a for the first time as a prognostic biomarker for recurrence in CRC patients. Similarly, Tsukamoto et al. [91] analyzed plasma exosomes from 326 CRC patients and identified miR-21 as a biomarker for predicting recurrence and prognosis in patients with Tumor-Node-Metastasis stages II, III, or IV colorectal cancer. Yan et al. collected 168 CRC patients for a retrospective cohort study. Compared with healthy controls, exosomal miR-6803-5p was significantly increased in serum samples from colorectal cancer patients, especially in CRC patients with advanced TNM or lymph node metastases and liver metastases had even higher levels of exosomal miR-6803-5p, and Cox regression analysis showed that patients with elevated levels had generally poorer OS and disease-free survival. Meanwhile, the area under the curve was 0.7399, indicating that exosomal miR-6803-5p has good predictive value [92].

Hu et al. [93] isolated and characterized exosomes from CRC patient sera and found that CAFs act by directly transferring exosomes to CRC cells, resulting in significantly miR-92a-3p levels in CRC cells increased, which further activates the Wnt/β-catenin signaling pathway and inhibits mitochondrial apoptosis by directly inhibiting FBXW7 and MOAP1, thereby contributing to cell stemness, EMT and chemoresistance in colorectal cancer. Suppression of serum of exosome leads to high expression of exosomal miR-92a-3p which effectively inhibits the development of metastasis and chemoresistance in colorectal cancer patients. Interestingly, PD-L1 is often seen at the infiltration edge of these TAMs, which may affect the infiltration of T cells [94], and immunotherapy against TAMs can also synergistically enhance the efficacy of immunotherapy and immune checkpoint inhibitors, however the relevant signaling pathways and specific mechanisms involved require further study for better understanding. Moreover, the combination of targeted macrophages and chemotherapy has shown significant reduction in tumor burden [95]. In recent studies, Tao et al. demonstrated that exosomal miR-208b is a non-invasive marker for predicting FOLFOX sensitivity in CRC. miR-208b is well delivered into recipient T cells by targeting PDCD4 to promote Treg expansion, further leading to tumor growth and oxaliplatin resistance [96] (Table 1). Therefore, it is expected that drugs can target the expression of tumor cell-derived exosomes and inhibit M2 polarization of macrophages or reprogram macrophages to M1 type, benefiting more CRC patients.

**Table 1:** Exosomal miRNAs as prognostic molecular markers

Cancer	Exosomal miRNA	Sources of miRNA	Results	Reference
Colorectal cancer	miR-155-5p	M2 macrophage	Enhance the proliferation of CD3 T cells and IFN-γ T cells, significantly reduce the expression level of IL-6, and lead to immune escape of colon cancer.	[25]
Colon cancer	miR-183-5p	CC cell	Mediating AKT/NF-κB pathway to accelerate CC progress.	[26]
Colorectal cancer	miR-1249-5p,	CRC cell	Inhibit the expression of TP53 in fibroblasts and promote the progress of cancer.	[27]
	miR-6737-5p			
	miR-6819-5p			
Colorectal cancer	miR-6825-5p	CRC cell	Up-regulating the expression of CXCR3 leads to the polarization of M2 and promotes the progress of CRC.	[28]
Colorectal cancer	miR-106-b	CRC cell	Inhibit the expression of PDCD4 in macrophages, activate PI3K/AKT/mTOR signaling pathway, and induce the polarization of M2 macrophages, thus promoting cancer metastasis.	[31]
Pancreatic cancer	miRNA-320a	CAFs	Regulation of PTEN/PI3Kγ signal transduction changes the phenotype of macrophages and accelerates the malignant development of pancreatic cancer.	[32]
Gastric cancer	miR-16-5p	M1 macrophage	Targeting PD-L1 triggered the immune response of T cells.	[33]
Colorectal cancer	miR-100,	Serum	Drug resistance.	[39]
	miR-92a,			
	miR-16,			
	miR-30e,			
	miR-144-5p			
Colorectal cancer	miR-625-3p	CAFs	Inhibition of the CELF2/WWOX pathway promotes the migration and invasion of CRC cells, EMT, and chemotherapy resistance.	[40]
Pancreatic cancer	miR-106-b	CAFs	The activation niche of CAF in fibroblasts before lung metastasis was induced, which led to the activation of NF-κB signal transduction.	[41]
Liver cancer	miR-1247-3p	Liver cancer cell	Drug resistance.	[42]
Colorectal cancer	miR-25-3p,	CRC cell	Activation of the PI3K/AKT signaling pathway regulates PTEN-induced M2 polarization of macrophages, significantly promotes EMT and VEGF, and induces CRLM.	[73]
	miR-130b-3p,			
	miR-425-5p			
Colorectal cancer	miR-21-5p,	CRC cell	Regulating PTEN/AKT can also regulate the SOCS1/STAT1 signaling pathway and significantly inhibit the expression of CD8+ T cells mediated by TAMs.	[76]
	miR-200a			
Colorectal cancer	miR-934	CRC cell	Down-regulating PTEN expression and activating PI3K/AKT signaling pathway can induce the polarization of M2 macrophages and promote the progress of CRC.	[77]
Colorectal cancer	miR-19a	Serum	Malignant prediction.	[90]
Colorectal cancer	miR-21	Serum	Malignant prediction.	[91]
Colorectal cancer	miR-6803-5p	Serum	Malignant prediction.	[92]
Colorectal cancer	miR-92a-3p	Serum	Activating the Wnt/β-catenin signaling pathway and inhibiting mitochondrial apoptosis by directly inhibiting FBXW7 and MOAP1 are helpful to the resistance of EMT and chemotherapy drugs in colorectal cancer.	[93]
Colorectal cancer	miR-208b	Serum	Drug resistance, proliferation.	[96]

The current research on exosomes is still in its early stages, and there are several challenges to consider. Technicians need to ensure the purity and quality of exosomes, which requires strict examination and optimization of extraction methods. Safety assessments are also crucial due to potential biological activity exosomes and their impact on tumor progression [97]. Variability in exosomes from different sources can affect study results, so it is important to explore specific molecular markers and develop personalized experimental scheme. Additionally, the polarization of macrophages in response to exosome complex, with both M1 and M2 subtype being influenced. but both are stimulated to polarize in different environmental conditions. Exosomes can promote the change of the M1 subtype of macrophages and initiate an immune response to enhance anti-tumor efficacy; On the other hand, exosomes can also activate the polarization of M2 macrophages and accelerate tumor progression. At present, it is impossible to ascertain that exosomes in tumor patients accurately mediate the specific phenotype of macrophages. Finally, we still do not fully understand the specific ways and mechanisms of exosomes miRNA mediating macrophage polarization, and how to avoid toxic and side effects on target organs, especially when exosomes deliver anti-tumor drugs, and a series of problems need to be further explored.

## Conclusion

In this article, the potential of exosomal miRNA-mediated macrophage polarization in diagnosing and treating CRC is discussed. It also mentions its application in other malignancies like lung cancer, cervical cancer, and urinary system tumors. Exosomes have advantages as diagnostic or prognostic markers due to their wide presence and stability in body fluids. They can also impact drug resistance by reducing exosome miRNA expression. Targeting tumor cell-derived exosomes to inhibit M2 macrophage polarization or reprogram them to M1 type could benefit more CRC patients. However, further exploration is still needed. The review searched for relevant literature on exocrine miRNA-mediated macrophages in tumors using PubMed.

## Data Availability

Data sharing is not applicable to this article as no new data were created or analyzed in this study.
